# Assessment of *SLX4* Mutations in Hereditary Breast Cancers

**DOI:** 10.1371/journal.pone.0066961

**Published:** 2013-06-26

**Authors:** Sohela Shah, Yonghwan Kim, Irina Ostrovnaya, Rajmohan Murali, Kasmintan A. Schrader, Francis P. Lach, Kara Sarrel, Rohini Rau-Murthy, Nichole Hansen, Liyng Zhang, Tomas Kirchhoff, Zsofia Stadler, Mark Robson, Joseph Vijai, Kenneth Offit, Agata Smogorzewska

**Affiliations:** 1 Clinical Genetics Service, Department of Medicine, Memorial Sloan-Kettering Cancer Center, New York, New York, United States of America; 2 Laboratory of Genome Maintenance, The Rockefeller University, New York, New York, United States of America; 3 Department of Epidemiology and Biostatistics, Memorial Sloan-Kettering Cancer Center, New York, New York, United States of America; 4 Department of Pathology, Memorial Sloan-Kettering Cancer Center, New York, New York, United States of America; 5 Human Oncology and Pathogenesis Program, Memorial Sloan-Kettering Cancer Center, New York, New York, United States of America; 6 Weill Cornell College of Medicine, New York, New York, United States of America; IFOM, Fondazione Istituto FIRC di Oncologia Molecolare, Italy

## Abstract

**Background:**

*SLX4* encodes a DNA repair protein that regulates three structure-specific endonucleases and is necessary for resistance to DNA crosslinking agents, topoisomerase I and poly (ADP-ribose) polymerase (PARP) inhibitors. Recent studies have reported mutations in *SLX4* in a new subtype of Fanconi anemia (FA), FA-P. Monoallelic defects in several FA genes are known to confer susceptibility to breast and ovarian cancers.

**Methods and Results:**

To determine if *SLX4* is involved in breast cancer susceptibility, we sequenced the entire *SLX4* coding region in 738 (270 Jewish and 468 non-Jewish) breast cancer patients with 2 or more family members affected by breast cancer and no known *BRCA1* or *BRCA2* mutations. We found a novel nonsense (c.2469G>A, p.W823*) mutation in one patient. In addition, we also found 51 missense variants [13 novel, 23 rare (MAF<0.1%), and 15 common (MAF>1%)], of which 22 (5 novel and 17 rare) were predicted to be damaging by Polyphen2 (score = 0.65–1). We performed functional complementation studies using p.W823* and 5 *SLX4* variants (4 novel and 1 rare) cDNAs in a human *SLX4*-null fibroblast cell line, RA3331. While wild type *SLX4* and all the other variants fully rescued the sensitivity to mitomycin C (MMC), campthothecin (CPT), and PARP inhibitor (Olaparib) the p.W823* *SLX4* mutant failed to do so.

**Conclusion:**

Loss-of-function mutations in *SLX4* may contribute to the development of breast cancer in very rare cases.

## Introduction

Genomic instability is a common hallmark of cancer cells and includes severe alterations of chromosome number or structure, known as chromosome instability (CIN) [1]. Patients with mutations in the critical DNA repair genes *BRCA1* [MIM 113705] or *BRCA2* [MIM 600185], exhibit a high level of CIN as well as hypersensitivity to chemicals that induce DNA double-strand breaks, such as inhibitors of poly(ADP-ribose) polymerase (PARP) [2]. Based on these findings, it was proposed that germline mutation of any genes that interact with *BRCA1/2*, or act in the same DNA repair pathway, may increase cancer susceptibility [3]. Sequencing of candidate genes from breast cancer patients revealed that monoallelic germline mutations of DNA repair genes *BRIP1* [MIM 605882], *PALB2* [MIM 610335], *ATM* [MIM 607585], *CHK2* [MIM 604373], *RAD51C* [MIM 602774] and *RAD51D* [MIM 602954] are associated with predisposition to hereditary breast cancer [3]–[5].

Biallelic mutations of some of these hereditary breast cancer susceptibility genes, namely *BRCA2/FANCD1, BRIP1/FANCJ, PALB2/FANCN* and *RAD51C/FANCO,* have been identified in patients with Fanconi anemia (FA, [MIM 227650]) [6]. FA is a rare recessive genetic disorder characterized by CIN, progressive bone marrow failure, congenital abnormalities, and predisposition to leukemia and solid tumors [7]. To date, biallelic mutations of 15 genes have been identified in FA patients. They encode eight proteins (FANC-A, -B, -C, -E, -F, -G, -L and -M), which form the FA core complex, two FA proteins (FANCD2 and FANCI), which form the ID complex and five proteins (FANCD1/BRCA2, FANCN/PALB2, FANCJ/BRIP1, FANCO/RAD51C and SLX4/FANCP) which are downstream effectors of the pathway [8]. Cell lines derived from FA patients display enhanced sensitivity to DNA cross-linking agents such as 1,2∶3,4-diepoxybutane (DEB), mitomycin C or cisplatin, and the FA pathway has been proposed to recognize and repair interstrand DNA crosslinks (ICLs). Activation of the pathway depends on multiple phosphorylation events and ubiquitination of FANCD2 and FANCI by the FANCL, a catalytic component of the FA core complex [9], [10]. Ubiquitinated FANCD2 and FANCI form a heterodimer and localize to the sites of DNA damage, leading to downstream ICL repair steps, including incision, translesion synthesis and homologous recombination [10], [11].

Recently, biallelic mutations of *SLX4/FANCP* [MIM 613278]/[MIM 613951] have been identified in patients with a new sub-type of FA, termed FA-P [12], [13]. Initially identified as one of the phosphorylation targets for ATM/ATR [14], SLX4 is a multi-domain scaffold protein, which interacts with at least three nucleases including SLX1, XPF-ERCC1 and MUS81-EME1 [13]–[16]. Biochemical assays showed that the SLX4-SLX1 complex displays Holliday junction resolvase activity and enhances the activity of XPF-ERCC1 and MUS81-EME1. In addition to nuclease interacting domains, SLX4 also contains two well-conserved ubiquitin binding zinc finger (UBZ) motifs and the BTB/POZ domain; however, the functional roles of these domains are not known. FA-P cell lines show ICL sensitivity and may also display topoisomerase I and PARP inhibitor sensitivity depending on the *SLX4* mutation [12], [17].

Monoallelic germline alterations of all previously identified downstream effectors in the FA pathways predispose to breast cancer, and the phenotype of patient cell lines is consistent with *SLX4* being essential for DNA repair, which led to our hypothesis that monoallelic germline mutations in *SLX4* might predispose carriers to breast cancer.

Over the last year, five studies have investigated the role of *SLX4* in familial *BRCA1/2* mutation-negative breast cancer cases. The first study reported 23 known and 4 novel missense mutations in 52 patients (28 German and 24 Byelorussian) [18]. In the second study, consisting of 526 patients from Italy, the investigators found 46 novel variants [19], of which 29 were missense, 14 were silent, two were intronic, and one was a 3-bp in-frame deletion. Only one of the 29 novel missense variants was predicted *in silico* to be pathogenic. In another study, *SLX4* was sequenced in 94 Spanish *BRCA*-negative patients [20]. Seven novel variants were not present in controls. The functional significance of these variants was not evaluated. In addition, Bakker et. al, identified 39 missense variants and one splice site mutation variant (c.2013+2T>A) in 729 BRCA-negative cases. Functional analysis of selected four missense variants using mitomycin C-induced growth inhibition did not show any loss of function. The splice site mutation was shown to result in skipping of exon 8, and was predicted to cause a premature stop codon in exon 9. The transcript from the mutant allele was expressed at lower levels than the wild type allele. The truncated form was not directly tested in complementation assays [21]. In a more recent study with 486 index cases from *BRCA1/2* mutation-negative breast and/or ovarian cancer families, de Garibay et. al. identified a truncating mutation (p.Glu1517*) and a missense mutation (p.Arg372Trp), predicted to be pathogenic by *in silico* analysis [22]. However, neither of these two mutations were tested functionally.

Here we present our SLX4 sequencing results in 738 *BRCA1/2* mutation-negative breast cancer patients and a functional analysis of select *SLX4* variants.

## Materials and Methods

### DNA Samples

Genomic DNA was extracted from peripheral blood of *BRCA1/2* mutation-negative breast cancer patients ascertained by the Clinical Genetics Service at Memorial Sloan-Kettering Cancer Center (MSKCC) between 1997 to 2011, following participant written consent and with MSKCC institutional review board approval. Previous *BRCA1/2* mutation testing included Ashkenazi founder mutation screening (136 samples), *BRCA1* and *BRCA2* full sequencing (381 samples) and gene sequencing plus rearrangement analysis (221 samples). DNA was extracted using Qiagen Gentra Puregene kit for extraction of whole EDTA anticoagulated blood (QIAGEN, Düsseldorf, Germany) according to the manufacturer's protocol and stored at the Diagnostic Molecular Genetics facility at MSKCC. Tumor tissue for the patient with a novel nonsense (c.2469G>A, p.W823*) mutation was obtained from the Tissue Procurement Service at MSKCC. DNA was isolated using Qiagen DNeasy Blood and Tissue kit (QIAGEN, Düsseldorf, Germany).

### PCR Amplification and Sequencing

The entire coding region and exon-intron boundaries of the *SLX4* gene were sequenced. Primers were designed using Primer3 [23] and M13 tags were added to facilitate Sanger sequencing. PCR reactions were carried out in 384 well plates, in an Eppendorf Mastercycler ep384 thermal cycler, using a touchdown PCR protocol with Kapa2G Fast HotStart Taq (Kapa Biosystems, Cape Town, South Africa). The touchdown PCR method consisted of: 1 cycle of 95°C for 5 min; 3 cycles of 95°C for 30 sec, 64°C for 15 sec, 72°C for 30 sec; 3 cycles of 95°C for 30 sec, 62°C for 15 sec, 72°C for 30 sec; 3 cycles of 95°C for 30 sec, 60°C for 15 sec, 72°C for 30 sec; 37 cycles of 95°C for 30 sec, 58°C for 15 sec, 72°C for 30 sec; 1 cycle of 70°C for 5 min. Templates were purified using AMPure (Beckman Coulter Genomics, Beverly, MA). The purified PCR reactions were split into two, and sequenced bidirectionally with M13 forward and reverse primers and Big Dye Terminator Kit v.3.1 (Applied Biosystems, Foster City, CA), at Beckman Coulter Genomics. Dye terminators were removed using the CleanSEQ kit (Beckman Coulter Genomics), and sequence reactions were run on ABI PRISM 3730xl sequencing apparatus (Applied Biosystems, Foster City, CA).

### Mutation Detection

Mutations were detected using an automated detection pipeline in the MSKCC Bioinformatics Core Service. Bi-directional reads and mapping tables (to link read names to sample identifiers, gene names, read direction, and amplicon) were subjected to a QC filter which excluded reads with an average phred score of <10 for bases 100–200. Passing reads were assembled against the reference sequences for each gene, containing all coding and UTR exons including 5Kb upstream and downstream of the gene, using command line Consed 16.0. [24]. Assemblies were passed on to Polyphred 6.02b [25] which generated a list of putative candidate mutations, and to Polyscan 3.0 [26] which generated a second list of putative mutations. The lists were merged together into a combined report, and the putative mutation calls were normalized to “+” genomic coordinates and annotated. To reduce the number of false positives generated by the mutation detection software packages, only mutations supported by at least one bi-directional read pair and at least one sample mutation called by Polyphred were considered and included in the final candidate list.

All putative mutations were confirmed by a second PCR and sequencing reaction. All traces for mutation calls were manually reviewed.

### Plasmids

A C-terminal deletion mutant of *SLX4* (for the expression of SLX4 W823*) was amplified by PCR using the wild-type *SLX4* cDNA (a kind gift from the Harper Lab, Harvard Medical School, Boston, MA). All other *SLX4* point mutation variants were generated with the QuikChange II XL Site-Directed Mutagenesis kit (Agilent Technologies) using the wild-type *SLX4* cDNA template.

### Cell Culture

Human fibroblast cell lines were grown in DMEM (Invitrogen) supplemented with 15% fetal bovine serum (HyClone, Thermo Scientific), 100 units of penicillin per milliliter and 0.1 mg of streptomycin per milliliter, nonessential amino acids, and 1 times GlutaMAX (Invitrogen). Fibroblasts were cultured in a 3% oxygen incubator. Human fibroblasts cell lines were transformed by HPV E6 and E7 proteins and immortalized with a catalytic subunit of human telomerase (hTERT) as indicated in the text.

### SLX4 Mutation Functional Analysis

A total of 3.0×10^4^ cells were plated in each well of a 6-well plate in triplicate. At 24 hours later, MMC (Sigma-Aldrich, M4287), CPT (Sigma-Aldrich, C9911), or a PARP inhibitor, Olaparib (O-9210, LC Laboratories) was added at the final concentration from 0–100 nM for MMC, 0–16 nM for CPT, or 0–50 µM for Olaparib. For PARP inhibitor sensitivity assay, drug-containing medium was replaced every two days. After 8 days in culture, cell numbers were counted with Z2 Coulter counter (Beckman Coulter). The cell numbers at each dose of drug was divided by the cell number in the untreated sample to calculate the percent survival.

## Results

We sequenced all exons and exon-intron boundaries of the *SLX4* gene in 738 (270 Jewish and 468 non-Jewish) breast cancer patients with *BRCA1/2* mutation-negative breast cancer and a family history of breast cancer with two or more additional affected individuals in the family. Probands consented to an institutional review board-approved protocol allowing use of specimens for genetic research. Patient age at the time of diagnosis ranged from 22 to 89 years (mean 60 years) and their ethnicities were: Caucasian (n = 704, 95%), Black/African descent (n = 13, 2%), White Hispanic (n = 15, 2%), and Asian/Far-East/Indian Subcontinent (n = 7, 1%). The cohort was enriched for patients of Jewish ancestry (n = 270, 37%).

**Figure 1:**
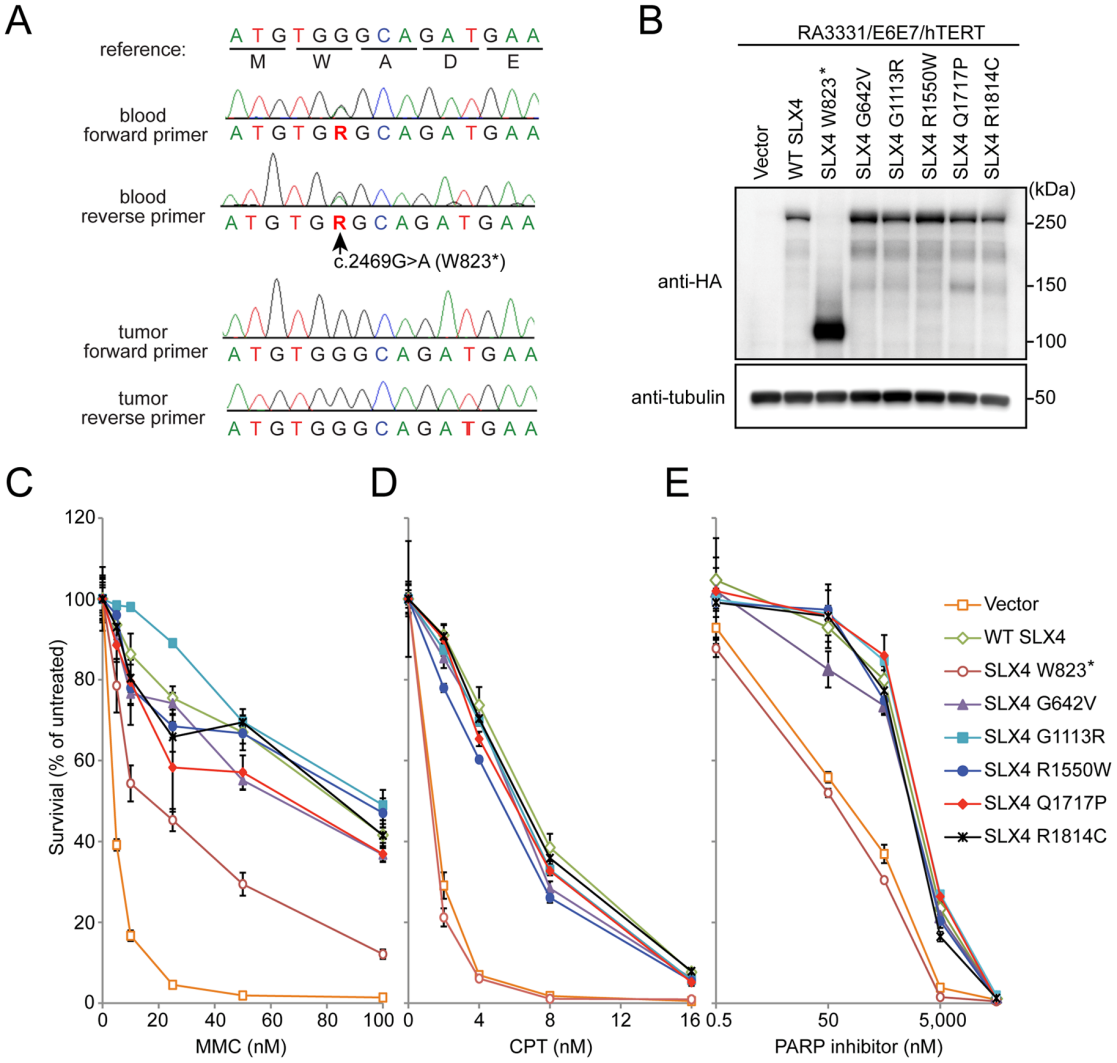
Identification of the nonsense*SLX4* variant and evaluation of pathogenicity of the *SLX4* variants by complementation assay. (A) Sanger sequence traces for DNA extracted from whole blood and breast tumor of the patient with the c.2469G>A (p.W823*) *SLX4* mutation (NCBI Refseq NM_032444.2). (B) Anti-HA immunoblot showing expression of HA-tagged SLX4 variants in RA3331/E6E7/hTERT cells. Alpha-tubulin serves as loading control. (C)∼(E) Cell survival assays of the RA3331/E6E7/hTERT cell lines expressing indicated SLX4 variants in response to MMC (C), CPT (D) and the PARP inhibitor, Olaparib (E). RA3331/E6E7/hTERT fibroblast cell lines expressing empty vector, wild type SLX4 and indicated SLX4 variants were treated in triplicate with increasing concentrations of MMC (0–100 nM), CPT (0–16 nM) and Olaparib (0–50 µM). After 8 days in culture, the cell number was determined using a Coulter counter. The number of cells at each drug concentration was divided by the number of cells in the untreated sample to calculate the percentage of cell survival. The error bars indicate standard deviations based on three replicates.

We found 51 missense variants and 1 truncating (c.2469G>A, p.W823*) mutation (Figure 1A and Table S1). Thirty eight of the missense variants have been previously characterized in the 1000 Genome [27], dbSNP databases [28], and Exome Variant Server (Exome Variant Server, NHLBI Exome Sequencing Project (ESP), Seattle, WA May, 2012). Of these, 6 are common variants with minor allele frequency (MAF) ≥5% and 6 others have MAF ≥1% (source dbSNP). The remaining 26 known variants are rare (MAF<0.1%). Of the known SNPs, 9 (rs77306735, rs147826749, rs138615800, rs115694169, rs59939128, rs7863028, rs141567438, rs72778139, rs111738042) were also seen in the three previous *SLX4* mutation screens (frequency< = 0.01). Another interesting observation was the co-occurrence of two neighboring SNPs, c.2854G>A (rs59939128, MAF = 0.069) and c.2855C>T (rs78637028, MAF = 0.045), in 56 patients. Both these alleles are reported independently in the dbSNP database and have different allele frequencies. In contrast, these 2 variants were also reported to have the same allele frequency, in two of the three previous *SLX4* mutation screenings in *BRCA1/2* mutation-negative breast cancer patients (10 [n = 52] and 8 [n = 94]) [18], [20] and were found to co-occur in 42 out of 526 patients in the third study [19].

We used the Wilcoxon test to compare age-of-onset between patients with or without missense SNPs. We found that the median age-of-onset was 2 years lower (47 vs 49) in SNP carriers vs non-carriers (p = 0.02). Of the 13 novel missense variants, five were predicted to be deleterious by Polyphen2 and SIFT. None of these five variants or the truncating mutation, p.W823*, were reported in the other *SLX4* breast cancer screens [18], [19], [20], [21], [22]. The patient carrying the p.W823* was diagnosed, in her 40s, with invasive lobular carcinoma of the breast, with metastatic carcinoma involving 15 lymph nodes. The breast tumor was positive for estrogen and progesterone receptors. The patient was of European descent and *BRCA1/2* mutation-negative. The patient’s mother, paternal grandmother, and father were diagnosed with breast cancer in her 60s, breast cancer in her 30s, and prostate cancer in his 60s, respectively. We sequenced the *SLX4* gene in the tumor from this patient and found loss of the mutant allele (Figure 1A).

We performed functional complementation assays in order to assess the pathogenicity of six *SLX4* variants (Figure 1B). For the complementation assays, we employed a human *SLX4*-null fibroblast cell line, RA3331 [12], [17], which has been immortalized with a catalytic subunit of human telomerase hTERT and transformed using HPV E6 and E7 proteins (RA3331/E6E7/hTERT) [12]. This cell line is sensitive to MMC, a topoisomerase I inhibitor (camptothecin, CPT) and a PARP inhibitor (Olaparib), (Vector, Figure 1C–E). We examined whether the *SLX4* cDNA harboring each of the novel variants could complement the sensitivity to each of the three drugs when introduced into this cell line. For the positive and negative control, wild-type *SLX4* cDNA and empty vector were transduced in the RA3331/E6E7/hTERT cell line. Expression level of the wild-type *SLX4* and the *SLX4* variants in the cell line was comparable (Figure 1B). Wild type SLX4 cDNA complemented MMC, CPT and Olaparib sensitivity of the human SLX4-null fibroblast cell line, RA3331/E6E7/hTERT. The five *SLX4* variants with missense mutations (G642V, G1113R, R1550W, Q1717P and R1814C) rescued the sensitivity to all three chemicals to the same level as wild-type *SLX4* implying that those missense mutations are unlikely to be pathogenic. However, we found that the truncated *SLX4* variant (W823*) failed to confer resistance to CPT and Olaparib and only partially rescued MMC sensitivity. The p.W823* SLX4 variant does not contain C-terminus of SLX4, which is critical for the interaction with MUS81-EME1 and SLX1. The lack of these interactions is responsible for the partial loss of SLX4 function seen in the complementation assay and we conclude that the p.W823* allele may be pathogenic.

## Discussion

We sequenced the entire coding region of the *SLX4* gene in 738 *BRCA1/2* mutation-negative familial breast cancer patients and found 26 previously known and 14 novel coding variants. These include a truncating mutation (c.2469G>A, p.W823*) in *SLX4*, which results in loss of function as demonstrated by our functional complementation assay. So far, three truncating *SLX4* mutations have been reported in 2,625 non-*BRCA1/2* breast cancer patients in all published work, including this study. One of them is E1517* [21], which, just like the truncation identified in the present study, removes domains responsible for interaction with MUS81 and SLX1 but leaves the XPF-interaction domain intact. Although the E1517* variant has not been directly tested in complementation assays, we have previously tested an SLX4 mutant consisting of amino acids 1 to 1520 [17] and found that it was still proficient in ICL repair, albeit at decreased level, but completely deficient for repair of CPT- or Olaparib-induced DNA damage. If the W823* and E1517* truncating variants are causative mutations and if these truncated SLX4 proteins are expressed, the differential sensitivity to ICL and other agents may indicate that the SLX4 function associated with MUS81 and/or SLX1 is essential for tumor suppression in the breast tissue. The splice site mutation variant (c.2013+2T>A) was shown to result in skipping of exon 8 and premature truncation of the protein and also resulted in lower expression of the transcript from the mutant allele [21]. The truncated protein, if at all expressed, would lack MUS81 and SLX1-interacting domains and would be expected to be fully deficient for repair of CPT and Olaparib-induced DNA damage. Our studies suggest that assessment of any SLX4 variants identified in breast cancers should include evaluation of their impact on sensitivity not only to ICL agents but also to CPT and Olaparib. Our previous studies showed that SLX4 is a multidomain protein that interacts with multiple nucleases that exert different functions in the cell [17]. We do not yet know which of these functions might be important for tumor suppression in the breast tissue so all known functions of SLX4 need to be tested to make prediction whether an identified variant might lead to loss of SLX4 function.

Sequencing of the *SLX4* gene in the tumor from the patient with the truncating mutation revealed loss of the mutant allele. This might mean that *SLX4* is not a breast cancer predisposition gene. However, loss of the mutant allele is not an unprecedented finding even for a bona fide breast cancer predisposition gene. In a recent study performed by King *et al*., 23 *BRCA*-linked breast tumors and 10 *BRCA*-linked prophylactic mastectomy (PM) specimens were analyzed for loss of the wild-type allele [29]. No loss of heterozygosity (LOH) or LOH involving the mutant allele was observed in a substantial fraction of pre-invasive and invasive breast carcinomas. The fraction without LOH or LOH of the mutant allele included 9 of 15 (60%) cases of ductal carcinoma-in-situ (DCIS) associated with invasive ductal carcinoma (IDC) and 11 of 22 (50%) IDCs. These results indicate that while LOH of the wild-type allele of the susceptibility gene is expected for tumorigenesis, this may not always be the case. Loss of the mutant *SLX4* allele in the tumor might also suggest that its presence may promote tumor progression by removing essential SLX4-dependent functions during the early stages of tumorigenesis. However, at the later stages the mutant allele becomes deleterious to the cells, and cells that remove it by LOH have better proliferative capacity. This is a testable hypothesis in a mouse model of *SLX4* loss. Another possibility is that *SLX4* might be epigenetically silenced in the tumor.

Overall, our study, in combination with other published work suggests that mutation in *SLX4* may be associated with increased risk of breast cancer in a very small number of familial breast cancers.

## Supporting Information

Table S1 *SLX4* variants found in *BRCA1/2* mutation negative familial breast cancer cases. ESP refers to NHLBI Exome Sequencing Project and 1KG is 1000 Genomes data.
(DOCX)
