# Supplementary material for: Assessment of SLX4 Mutations in Hereditary Breast Cancers
Source: PLoS One. 2013 Jun 26;8(6):e66961. doi: 10.1371/journal.pone.0066961 (PMC3694110; doi:10.1371/journal.pone.0066961)
Supplement: Table S1 — SLX4 variants found in BRCA1/2 mutation negative familial breast cancer cases. ESP refers to NHLBI Exome Sequencing Project and 1KG is 1000 Genomes data. (DOCX) [file pone.0066961.s001.docx]

Table S1*. SLX4* variants found in *BRCA1/2* mutation negative familial breast cancer cases. ESP refers to NHLBI Exome Sequencing Project and 1KG is 1000 Genomes data.

| cDNA | Type | Amino acid change | # of samples | Frequency in cohort | Polyphen prediction | rs number | MAF (dbSNP) | Source |
| --- | --- | --- | --- | --- | --- | --- | --- | --- |
| c.85C>T | Missense | p.R29C | 1 | 0.0013 | benign | rs144832924 | 0.001 | ESPs |
| c.86G>A | Missense | p.R29H | 5 | 0.0067 | benign | [rs149117119](http://www.ncbi.nlm.nih.gov/SNP/snp_ref.cgi?rs=149117119) | - | ESP |
| c.145G>A | Missense | p.E49K | 1 | 0.0013 | possibly damaging | rs139757085 | - | ESP |
| c.421G>T | Missense | p.G141W | 2 | 0.0027 | probably damaging | rs77306735 | 0 | 1KG |
| c.497A>C | Missense | p.Q166P | 3 | 0.0040 | benign | NA | - | - |
| c.590T>C | Missense | p.V197A | 2 | 0.0027 | benign | rs147826749 | 0.001 | ESP |
| c.610C>T | Missense | p.R204C | 55 | 0.0741 | probably damaging | rs79842542 | 0.059 | 1KG |
| c.710G>A | Missense | p.R237Q | 1 | 0.0013 | benign | rs138615800 | 0.007 | 1KG |
| c.1077G>T | Missense | p.K359N | 1 | 0.0013 | benign | rs149470704 | - | ESP |
| c.1153C>A | Missense | p.P385T | 6 | 0.0081 | benign | rs115694169 | 0.009 | 1KG |
| c.1156A>G | Missense | p.M386V | 30 | 0.0404 | benign | rs113490934 | 0.049 | 1KG |
| c.1243G>A | Missense | p.E415K | 4 | 0.0054 | possibly damaging | rs146021821 | - | ESP |
| c.1371T>G | Missense | p.N457K | 61 | 0.0822 | benign | rs74319927 | 0.051 | 1KG |
| c.1372A>G | Missense | p.K458E | 2 | 0.0027 | probably damaging | rs149126845 | - | ESP |
| c.1898G>A | Missense | p.G633D | 2 | 0.0027 | benign | rs1056085 | 0.009 | 1KG |
| c.1925G>T | Missense | p.G642V ‡ | 1 | 0.0013 | probably damaging | NA | - | - |
| c.2012T>C | Missense | p.L671S | 57 | 0.0768 | benign | rs77985244 | 0.06 | ESP |
| c.2305G>C | Missense | p.E769Q | 2 | 0.0013 | benign | rs150712805 | 0.001 | ESP |
| c.2359G>A | Missense | p.E787K | 3 | 0.0040 | possibly damaging | [rs140600202](http://www.ncbi.nlm.nih.gov/SNP/snp_ref.cgi?rs=140600202) | - | ESP |
| c.2437G>A | Missense | p.E813K | 1 | 0.0013 | probably damaging | NA | - | - |
| c.2469G>A | Nonsense | p.W823* | 1 | 0.0013 | NA | NA | - | - |
| c.2824G>C | Missense | p.E942Q | 6 | 0.0081 | probably damaging | rs114014006 | 0.02 | 1KG |
| c.2854G>A | Missense | p.A952T | 3 | 0.0040 | benign | rs59939128 | 0.058 | 1KG |
| c.2854G>A | Missense | p.A952M | 56 | 0.0755 | probably damaging | rs59939128 | 0.058 | 1KG |
| c.2855C>T | Missense | p.A952M | 56 | 0.0755 | probably damaging | rs78637028 | 0.033 | 1KG |
| c.2924C>T | Missense | p.P975L | 7 | 0.0094 | benign | rs114472821 | 0.008 | 1KG |
| c.2975G>A | Missense | p.G992E | 1 | 0.0013 | possibly damaging | rs139287784 | 0.001 | ESP |
| c.3019C>A | Missense | p.Q1007K | 3 | 0.0040 | benign | rs138798067 | - | ESP |
| c.3034A>G | Missense | p.R1012G | 1 | 0.0013 | benign | NA | - | - |
| c.3118C>T | Missense | p.P1040S ‡ | 1 | 0.0013 | benign | NA | - | - |
| c.3178C>T | Missense | p.R1060W | 8 | 0.0108 | benign | [rs144273492](http://www.ncbi.nlm.nih.gov/SNP/snp_ref.cgi?rs=144273492) | - | ESP |
| c.3337G>C | Missense | p.G1113R | 1 | 0.0013 | benign | NA | - | - |
| c.3365C>T | Missense | p.P1122L | 104 | 0.1402 | benign | rs714181 | 0.208 | 1KG |
| c.3368C>A | Missense | p.S1123Y | 1 | 0.0013 | possibly damaging | rs144647122 | - | ESP |
| c.3662C>T | Missense | p.A1221V | 56 | 0.0755 | benign | rs3827530 | 0.041 | 1KG |
| c.3676C>T | Missense | p.R1226W | 2 | 0.0027 | probably damaging | rs142008398 | - | ESP |
| c.3812C>T | Missense | p.S1271F | 81 | 0.1092 | probably damaging | rs3810813 | 0.088 | 1KG |
| c.3913G>A | Missense | p.A1305T | 1 | 0.0013 | benign | NA | - | - |
| c.4057C>A | Missense | p.H1353N | 1 | 0.0013 | benign | [rs142205392](http://www.ncbi.nlm.nih.gov/SNP/snp_ref.cgi?rs=142205392) | - | ESP |
| c.4261A>T | Missense | p.I1421F | 2 | 0.0027 | possibly damaging | [rs141567438](http://www.ncbi.nlm.nih.gov/SNP/snp_ref.cgi?rs=141567438) | - | ESP |
| c.4403G>A | Missense | p.R1468H | 1 | 0.0013 | benign | NA | - | - |
| c.4409C>T | Missense | p.P1470L | 2 | 0.0013 | possibly damaging | rs72778139 | - | ESP |
| c.4523C>T | Missense | p.S1508L | 4 | 0.0027 | probably damaging | rs112694849 | - | Bushman |
| c.4597G>A | Missense | p.A1533T ‡ | 1 | 0.0013 | benign | NA | - | - |
| c.4600G>A | Missense | p.G1534S | 3 | 0.0040 | benign | rs78770603 | 0.049 | 1KG |
| c.4649G>A | Missense | p.R1550Q | 1 | 0.0013 | benign | NA | - | - |
| c.4648C>T | Missense | p.R1550W | 3 | 0.0040 | benign | rs77021998 | 0.005 | 1KG |
| c.5029C>T | Missense | p.P1677S | 2 | 0.0027 | benign | rs7196345 | 0.009 | 1KG |
| c.5146T>A | Missense | p.S1716T | 2 | 0.0027 | probably damaging | rs75182789 | 0.032 | 1KG |
| c.5150A>C | Missense | p.Q1717P | 1 | 0.0013 | probably damaging | NA | - | - |
| c.5189C>T | Missense | p.S1730F * | 1 | 0.0013 | possibly damaging | NA | - | - |
| c.5440C>T | Missense | p.R1814C | 1 | 0.0013 | probably damaging | NA | - | - |
| c.5501A>G | Missense | p.N1834S | 10 | 0.0135 | benign | rs111738042 | 0.005 | 1KG |

‡ novel missense variants identified only in the patients of Jewish ancestry
